# Unraveling the role of social support in eating behavior among children and adolescents in Shanghai, China: exploring the mediating role of self-efficacy and the moderating influence of BMI and weight concern

**DOI:** 10.3389/fnut.2024.1411097

**Published:** 2024-07-04

**Authors:** Shuoyuan Tan, Rui Yang, Gulqihra Abdukerima, Yimin Xu, Lihong Zhu, Bin Xu, Wenwei Shen, Lixin Song, Bing Ji, Zhaoxin Wang, Chen Chen, Jianwei Shi

**Affiliations:** ^1^School of Public Health, Shanghai Jiao Tong University School of Medicine, Shanghai, China; ^2^East China Model High School, Shanghai, China; ^3^Shanghai No.1 Middle School, Shanghai, China; ^4^Jing’an No.2 Central Primary School, Shanghai, China; ^5^Shanghai Jing’an Experimental Primary School, Shanghai, China; ^6^Shanghai Jing’an District Jiangning Road Community Health Service Center, Shanghai, China; ^7^The First Affiliated Hospital, Hainan Medical University, Haikou, Hainan, China; ^8^School of Management, Hainan Medical University, Haikou, Hainan, China; ^9^Department of General Practice, Yangpu Hospital, Tongji University School of Medicine, Shanghai, China; ^10^Department of Social Medicine and Health Management, School of Public Health, Shanghai Jiao Tong University School of Medicine, Shanghai, China

**Keywords:** eating behavior, self-efficacy, social support, weight concern, obesity

## Abstract

**Objective:**

This study explores the intricate relationship between social support and eating behaviors in children and adolescents, considering the mediating role of eating self-efficacy and the moderating effects of body mass index (BMI) and weight concern.

**Methods:**

Data from 1986 primary and secondary school students aged 8 to 17 in Shanghai, China, were analyzed using moderated mediation analysis.

**Results:**

The results demonstrate a robust positive association between social support and eating self-efficacy, particularly prominent among individuals with low BMI (effect = 0.506, 95% CI [0.376, 0.636]). Moreover, the study highlights that eating behavior is influenced not only by eating self-control (β = −0.054, 95% CI [−0.062, −0.046]) but also by the interaction term between individuals’ perceptions of their body weight (β = −0.0008, 95% CI [−0.0015, −0.0001]).

**Conclusion:**

Eating self-efficacy serves as a mediator in the relationship between social support and eating behavior, modulated by BMI and weight concern. Importantly, high weight concern significantly strengthens the mediating effect of eating self-efficacy on the relationship between social support and eating behavior, regardless of BMI.

## 1 Introduction

In recent decades, obesity among children and adolescents has surged, with the prevalence rate increasing approximately eightfold (1). This escalation is closely tied to unhealthy eating behaviors, including the consumption of high-energy-dense foods and beverages (2). A study involving Bangladeshi adolescents aged 13–19 revealed that the likelihood of obesity among those with unhealthy eating behaviors was 1.634 (95% CI 1.495–1.786) compared to those with healthy eating behaviors (3). Despite numerous intervention studies targeting eating behaviors, treatments often fail shortly after initiation. This failure is largely attributed to our limited understanding of the psychological factors behind unhealthy eating behaviors and a flawed assumption that teenagers will readily modify their unhealthy behaviors solely in response to persuasion from teachers, parents, and other sources (4).

Eating behaviors have been shown to be associated with individual characteristics (e.g., body mass index) (5), interpersonal interactions (e.g., social support) (6), and an individual’s perceived confidence (i.e., self-efficacy) (7). Recent studies have suggested that social support serves as a positive impetus for healthy eating (8, 9). Furthermore, social support and self-efficacy are significantly correlated and play crucial predictive roles in healthy eating behaviors, as validated in populations such as pregnant women (2), athletes (10) and middle-aged and elderly individuals (11). Previous studies consistently indicate that higher levels of social support can enhance health dietary habits through self-efficacy, resulting in reduced fat intake and increased consumption of fruits and vegetables. However, there is limited understanding regarding the association between social support and healthy eating behaviors among children and adolescents in middle-income countries.

Building upon the Theory of Planned Behavior and the Health Action Process Approach, this study posits that social support fosters intentions and subsequently impacts eating behavior through the mediation of self-efficacy. Social support is defined as individuals’ perception of the care, support, and assistance received from family members, friends, and others (9, 12).

Demonstrated as a protective factor, social support has consistently shown a positive association with eating self-efficacy. Eating self-efficacy, also known as self-efficacy in weight management, reflects one’s confidence in self-regulation to achieve healthy eating behaviors despite temptations (13). Higher levels of eating self-efficacy indicate stronger self-regulation, leading to healthier eating behaviors.

BMI can significantly influence the psychological well-being of adolescents. For example, a study has found that obese adolescents often experience higher levels of self-consciousness and inner sensitivity (14). Groshon demonstrated that individuals with high BMI may internalize stereotypes of lacking self-control, leading to a reduction in eating self-efficacy (15). To better explore the psychological states of youths with different BMIs and their potential impact on eating behaviors, this study incorporates BMI as a moderating variable between social support and eating self-efficacy. It hypothesizes that high BMI may weaken the effect of social support on eating self-efficacy.

It is further hypothesized that the influence of eating self-efficacy on eating behavior is moderated by weight concern, which refers to an individual’s perception of their body weight. The hypothesis is grounded in the concept that “people will have greater self-efficacy if they perceive stronger motivation and severity” (16), and is supported by research confirming the interrelationships among weight concern, self-efficacy, and eating behavior (17, 18). Eating self-efficacy may be significantly correlated with increased adoption of diet-promoting behaviors, particularly notable when individuals express heightened concern about their weight.

**FIGURE 1:**
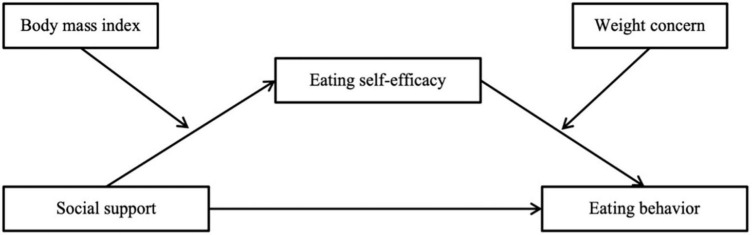
Conceptual framework of this study.

In this study, we present a structural equation model aimed at uncovering the intricate pathways connecting social support with healthy eating behavior, utilizing eating self-efficacy as a mediator and BMI and weight concern as moderators (see Figure 1). Our aim is to enhance comprehension of the psychological mechanisms through which social support influences changes in eating behavior among children and adolescents. By addressing current research disparities, our study provides crucial insights for the development of effective weight loss interventions in the future.

## 2 Materials and methods

### 2.1 Participants and procedures

The cross-sectional study, employing cluster random sampling, was conducted in Shanghai, China, from October to December 2023. Jing’an District was specifically chosen as the study site due to its central location within Shanghai and its representation of the city’s average economic status. Within this district, a neighborhood administrative office (street office) was randomly selected. Subsequently, all public primary and secondary schools with four or more enrollment classes within the jurisdiction of this selected neighborhood administrative office were identified and enlisted. Random numbers were then assigned to each eligible school, and using a randomized selection process, two middle schools and two primary schools were chosen from this pool of eligible institutions. To accommodate the cognitive levels of younger students, data collection in primary schools covered grades 3 to 5. Additionally, in recognition of the transition pressures associated with higher education, junior high schools encompassed grades 6 through 8, while senior high schools included grades 10 and 11. The study questionnaire consisted of 166-items. Following the principle of sample size determination, which dictates that the sample size should statistically cover ten times the number of items (19), a minimum sample size of 1660 was determined. Factoring in a 20% non-response rate, the target sample size was set at 2075.

Based on the research objectives, we developed a web-based information system comprising of six modules encompassing demographic information, physical activity, eating behavior, sleep patterns, psychological aspects and social support. After consent was obtained from the participants and legal guardians, all eligible students accessed the system with the assistance of the researchers to complete the questionnaire autonomously. Furthermore, a professional team conducted physical examinations on students, including measurements of height, weight, visual acuity, and other relevant indicators.

A total of 2106 students completed the questionnaire, yielding 1986 (94.30%) valid responses. Among these, 1056 (53.18%) were male, and 930 (46.83%) were female. The surveyed population comprised 670 primary school students, 660 junior high school students, and 656 senior high school students, with ages predominantly falling between 8 and 17 years.

### 2.2 Measures

#### 2.2.1 Anthropometric measures

Body weight (recorded to the nearest 0.1kg) and height (recorded to the nearest 0.5cm) were obtained during the physical examination. BMI values were then calculated using the formula: weight (kg)/height (m^2^).

#### 2.2.2 Social support

Social support was measured by the Social Support Appraisals (SS-A) scale (20), consisting of three subscales: support from family, friends, and others outside of family and friends. This scale, comprising 20 items rated on a 5-point Likert scale, has been extensively utilized in Chinese adolescents (21). A total score was calculated by summing these items, with higher scores indicating greater perceived social support. In the present study, the Cronbach’s α coefficient for the entire scale was 0.849.

#### 2.2.3 Eating self-efficacy

Participants’ eating self-efficacy was evaluated using the Weight Efficacy Lifestyle Questionnaire Short-Form (WEL-SF), originally developed by Matthew M. Clark (22) and revised by G. E. Ames (23). This 8-item questionnaire has been validated to assess self-efficacy judgments regarding eating behavior (24), with a higher total summed score indicating stronger eating self-efficacy. The questionnaire demonstrated strong psychometric properties in the current sample (Cronbach’s α = 0.937).

#### 2.2.4 Weight concern

Body weight concern was assessed using The Weight Concerns Scale (WCS) (25), a valid measure consisting of five items rated on a Likert-type scale ranging from 4 to 7 points (26). This scale was developed based on the observed strong association between eating disorder symptoms and concerns related to body weight, with higher scores indicating greater weight concerns. In our study, the Cronbach’s α coefficient for this scale was computed at 0.788.

#### 2.2.5 Eating behavior

The eating behavior questionnaire was adapted from the Chinese Adolescent Health-Related Behavior Survey (27). It comprises 10 items assessing the frequency of consumption of fruits, vegetables, dairy products, and unhealthy foods (snacks, sugar-sweetened beverages, desserts, and fried foods). Additionally, it calculates the frequency of breakfast consumption, dining at Western-style fast food restaurants and street vendors, and identifies whether there is selective eating behavior. Unhealthy eating behavior was indicated by a higher total score, the internal consistency of this data was α = 0.628.

### 2.3 Data analyses

Social support, eating self-efficacy, eating behavior, BMI, and weight concern were included in the analyses, with social support serving as the independent variable and eating behavior as the dependent variable. Spearman correlations were utilized to explore both the intra- and inter-relationships among the variables. We examined the distribution of variables and standardized weight concern, social support, eating self-efficacy, and BMI. SPSS Statistics Version 27.0, incorporating the PROCESS macro-instructional software Model 21 (28) with 5000 bootstrapped resamples, was employed to investigate whether eating self-efficacy mediates the relationship between social support and eating behavior, and whether this mediation is moderated by BMI and weight concern when adding gender as a covariate. A simple slope test was then used to examine the moderator effect (29).

## 3. Results

### 3.1 Descriptive analysis results

Table 1 presents the descriptive analysis and bivariate analysis of the variables under investigation. Participants exhibited a moderate to high level of social support (Mean = 50.030) and relatively high eating self-efficacy (Mean = 55.390), indicating a strong confidence in overcoming challenges associated with excessive eating. Additionally, a significant positive correlation was observed between social support and eating self-efficacy (*r* = 0.218, *p* < 0.001), suggesting that higher levels of social support were associated with greater eating self-efficacy. Furthermore, both social support (*r* = −0.222, *p* < 0.001) and eating self-efficacy (*r* = −0.033, *p* < 0.001) demonstrated significant negative correlations with eating behavior, indicating that higher scores in social support and perceived eating self-efficacy were associated with lower scores on eating behavior questionnaires and fewer unhealthy eating behaviors.

**TABLE 1:** Descriptive statistics and correlation analysis.

Variables	*M*	SD	Correlations				
			1	2	3	4	5
1. Social support	50.030	10.414	1.000	0.218***	−0.222***	−0.064**	−0.142***
2. Eating self-efficacy	55.390	22.334		1.000	−0.033***	−0.123***	−0.175***
3. Eating behavior	18.570	4.206			1.000	0.091***	0.025
4. Body mass index	20.417	5.752				1.000	0.276***
5. Weight concern	13.523	11.592					1.000

*N* = 1986; **p < 0.01, ***p < 0.001.

### 3.2 Moderated mediation analysis results

**FIGURE 2:**
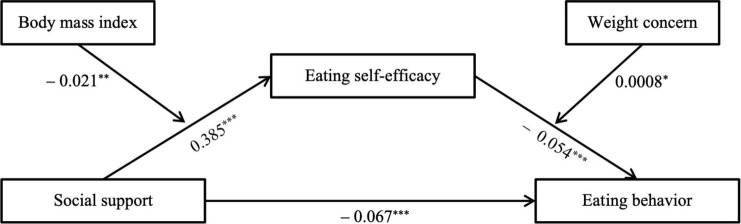
Moderated mediation model with indirect and direct effects of eating self-efficacy and social support on eating behavior. *Indicates significant paths: *p < 0.05, **p < 0.01, ***p < 0.001.

Table 2 displays the direct relations between social support, eating self-efficacy and eating behavior, as well as the indirect effects of social support on eating behavior mediated by eating self-efficacy, as moderated by BMI and weight concern. Figure 2 illustrates the results of the model testing, highlighting statistically significant paths (*p* < 0.05). The model significantly predicted eating behavior, R-squared = 0.13, *F* = 69.90, *p* < 0.001.

**TABLE 2:** Moderated mediation analysis results.

Direct effects	Eating self-efficacy (SE)			Eating behavior (EB)		
	β (SE)	β 95% CI	*T*-value	β (SE)	β 95% CI	*T*-value
Social support (SS)	0.385 (0.047)***	(0.292, 0.478)	8.125	−0.067 (0.009)***	(−0.085, −0.050)	−7.736
Body mass index (BMI)	−0.369 (0.103)***	(−0.571, −0.168)	−3.590			
SS × BMI	−0.021 (0.008)**	(−0.036, −0.006)	−2.674			
Eating self-efficacy (SE)				−0.054 (0.004)***	(−0.062, −0.046)	−13.159
Weight concern (WC)				−0.040 (0.008)	(−0.020, 0.110)	−0.484
SE × WC				−0.0008 (0.0003)*	(−0.0015, −0.0001)	−2.283
**Conditional indirect effects**				**Effect**	**95% CI**	
SS → SE → EB: low BMI, low WC				−0.023	(−0.031, −0.009)	
SS → SE → EB: low BMI, medium WC				−0.027	(−0.036, −0.011)	
SS → SE → EB: low BMI, high WC				−0.032	(−0.043, −0.013)	
SS → SE → EB: medium BMI, low WC				−0.017	(−0.024, −0.011)	
SS → SE → EB: medium BMI, medium WC				−0.021	(−0.027, −0.015)	
SS → SE → EB: medium BMI, high WC				−0.024	(−0.033, −0.016)	
SS → SE → EB: high BMI, low WC				−0.012	(−0.025, −0.006)	
SS → SE → EB: high BMI, medium WC				−0.014	(−0.031, −0.007)	
SS → SE → EB: high BMI, high WC				−0.017	(−0.035, −0.008)	

SE, standard error; 95% CI = 95% confidence interval. *p < 0.05, **p < 0.01, ***p < 0.001.

Social support exhibited a significant negative relationship with eating behavior (β = −0.067, *p* < 0.001), supporting the hypothesis that higher levels of social support are associated with healthier dietary patterns. Furthermore, increased social support was significantly linked to higher eating self-efficacy, moderated by BMI (β = 0.385, *p* < 0.001), while eating self-efficacy was significantly associated with healthy eating behavior moderated by weight concern (β = −0.054, *p* < 0.001). This suggests that social support indirectly promotes healthy eating behavior by enhancing eating self-efficacy. To be more specific, through eating self-efficacy, the impact of social support is particularly robust when BMI is low and weight concern is high (conditional indirect: −0.032, 95% CI from −0.043 to −0.013). Conversely, the indirect effect is minimal among adolescents with high BMI and low weight concern (conditional indirect: −0.012, 95% CI from −0.025 to −0.006). Moreover, high weight concern can mitigate the impact of high BMI, with the effect being amplified when both BMI and weight concern are high (conditional indirect: −0.017, 95% CI from −0.035 to −0.008).

### 3.3 Simple slope analysis results

**FIGURE 3:**
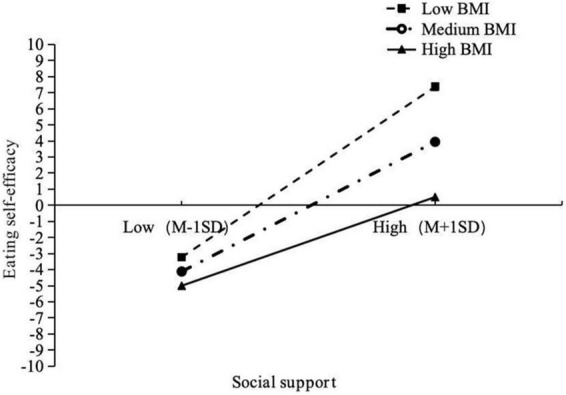
Simple slope analysis: relationship between social support and eating self-efficacy moderate by BMI.

To further elucidate the moderation effects, moderators were categorized by addition or subtraction of one standard deviation from the mean, and simple slope tests were carried out to explore the relationships (see Table 3). As depicted in Figure 3, with increasing BMI, the effect diminishes, suggesting that social support has a stronger influence on eating self-efficacy among adolescents with lower BMI. Specifically, when BMI is low (−1 SD), the effect is 0.506 (*p* < 0.001). When the value reaches the mean, the influence decreases to 0.385 (*p* < 0.001). Further increasing the moderator value to high (1 SD) results in a decreased influence of 0.265 (*p* < 0.001).

**TABLE 3:** Moderation analysis results.

Variables	Moderator	Values	Effect	95% CI	
				Lower limit	Upper limit
Social Support	Body mass index	Low (-1SD)	0.506***	0.376	0.636
		Medium (the mean)	0.385***	0.292	0.478
		High (+ 1SD)	0.265***	0.138	0.391
Eating self-efficacy	Weight concern	Low (-1SD)	−0.045***	−0.055	−0.035
		Medium (the mean)	−0.054***	−0.062	−0.046
		High (+ 1SD)	−0.063***	−0.075	−0.051

95% CI = 95% confidence interval. ***p < 0.001.

**FIGURE 4:**
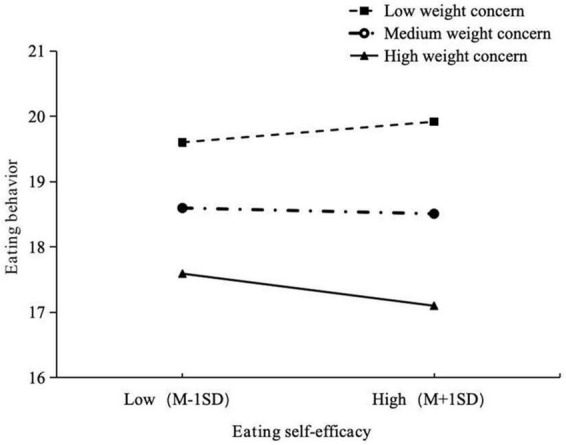
Simple slope analysis: relationship between eating self-efficacy and eating behavior moderate by weight concern.

The simple slope test examines the interaction between eating self-efficacy and weight concern on eating behavior (see Figure 4). Eating self-efficacy significantly contributes to healthy eating behavior to a greater extent when weight concern is high (effect = −0.063, *p* < 0.001) compared to when it is low (effect = −0.045, *p* < 0.001). Notably, individuals with high weight concern and low levels of eating self-efficacy exhibit elevated scores on the eating behavior questionnaire, indicating unhealthy eating behavior. These findings underscore the importance of a positive combination of eating self-efficacy and weight concern for fostering healthy eating behavior.

## 4 Discussion

To our knowledge, this study represents a pioneering attempt to introduce a conceptual model examining whether self-efficacy mediates the relationship between social support and eating behavior among children and adolescents in China. The findings from a substantial dataset support our hypothesis that self-efficacy mediates the association between social support and eating behavior, with BMI and weight concern moderating these effects.

The results revealed strong associations between social support, eating self-efficacy, and healthy eating behavior. Consistent with prior research (2, 13), individuals experiencing greater social support showed increased eating self-efficacy, leading to enhanced healthy eating behaviors. Furthermore, compared to adolescents with high BMI, those with low BMI exhibited stronger indirect effects. In other words, high social support brings about stronger eating self-efficacy among adolescents with low BMI, thereby improving their healthy eating behavior. A cross-sectional study in Finnish adults also found that a weak healthy self-efficacy was associated with increasing BMI, and perceived healthy self-efficacy correlated with a healthy food pattern (30). Additionally, the study revealed that regardless of BMI, social support significantly influenced healthy eating behavior through eating self-efficacy, particularly when weight concern was high. However, low eating self-efficacy in adolescents coupled with high weight concern paradoxically increased unhealthy eating behaviors. Previous research has underscored the link between weight concern and eating disorders, where heightened weight concern heightened the risk of binge eating (31). A plausible explanation is that heightened weight concern elevates the risk of binge eating, coupled with low eating self-efficacy, resulting in inadequate self-control and increased unhealthy eating behaviors. Overall, these findings highlight a combination of positive social support, eating self-efficacy, and weight concern in improving healthy eating behaviors and facilitating weight loss.

This article not only holds theoretical significance by addressing a research gap concerning the mechanisms of social support and dietary behavior among children and adolescents but also offers practical implications. It urges policymakers to integrate support from various sectors of society to develop specialized solutions for addressing the increasingly serious issue of childhood and adolescent obesity. Social support is consistently believed to contribute to the development of healthy eating behaviors, including the establishment of supportive environments and the provision of public weight-loss programs (32). Additionally, study by Gitta et al. emphasize that it is not solely the provision of social investment that matters, but also the enhancement of the public’s subjective perception of the usefulness of social resources and support mechanisms, which further aids in the development of healthy eating behaviors (8). Research indicates that self-efficacy plays a crucial role in fostering healthy dietary behaviors (33). Moreover, parental dietary practices, feeding methods, and communication styles with their children can influence their children’ s autonomy regarding eating behaviors and food choices (34, 35). Hence, collaborative efforts among families, schools, and community health centers are essential to conduct dietary self-regulation training for obese and overweight students, imparting knowledge on healthy eating, enhancing awareness, establishing correct self-image perceptions and weight perceptions. While improving eating self-efficacy, it is important to recognize the potential health risks associated with high BMI, increase weight concern, and promptly assist overweight adolescents in achieving weight loss goals.

While this study advances our understanding of the associations between social support and eating behavior, it is important to recognize several limitations. Firstly, the study data are cross-sectional, which restricts the ability to establish definitive directional relationships among the variables. Variables in the model, particularly BMI, weight concern, and social support, are subject to change over time. Therefore, longitudinal studies are essential to accurately monitor these fluctuations and their impact on eating behavior. Secondly, due to cost considerations, self-reported eating behavior questionnaires were used, assessing the frequency of consumption of various food items rather than quantifying absolute amounts. This approach may introduce biases, and ideally, more comprehensive questionnaires or 24-h dietary recalls should be employed. Thirdly, this study did not account for the influence of parental food practices, which may introduce bias into the data. This underscores the importance of considering confounding variables in future research to ensure the accuracy of results. Finally, the limited generalizability of the study findings may be attributed to specific sample characteristics, as the study only included primary and secondary school students from Shanghai. Future research should include data from more economically disadvantaged areas to confirm the external validity of the results.

In conclusion, the present study suggests that eating self-efficacy serves as a valid mediator of the influence of social support on eating behavior among Chinese children and adolescents. This mediation effect varied depending on BMI and weight concern; individuals with a lower BMI exhibited stronger eating self-efficacy, while high weight concern facilitated the transformation of social support into healthier eating behaviors through eating self-efficacy.

## Data availability statement

The raw data supporting the conclusions of this article will be made available by the authors, without undue reservation.

## Ethics statement

The studies involving humans were approved by the Public Health and Nursing Research Ethics Committees affiliated to Shanghai Jiao Tong University School of Medicine (ref: SJUPN-20211). The studies were conducted in accordance with the local legislation and institutional requirements. Written informed consent for participation in this study was provided by the participants’ legal guardians/next of kin.

## Author contributions

ST: Data curation, Formal analysis, Writing – original draft. RY: Data curation, Writing – original draft. GA: Data curation, Writing – review and editing. YX: Resources, Writing – review and editing. LZ: Resources, Writing – review and editing. BX: Resources, Writing – review and editing. WS: Resources, Writing – review and editing. LS: Investigation, Writing – review and editing. BJ: Investigation, Writing – review and editing. ZW: Project administration, Writing – review and editing. CC: Investigation, Project administration, Resources, Writing – review and editing. JS: Funding acquisition, Investigation, Project administration, Resources, Writing – review and editing.

## References

[1] Nature Reviews Disease Primers. Child and adolescent obesity. *Nat Rev Dis Prim.* (2023) 9:25. 10.1038/s41572-023-00440-7 37202378

[2] Chang MW Schaffir J Brown R Wegener DT. Mediation by self-efficacy in the relation between social support and dietary intake in low-income postpartum women who were overweight or obese. *Appetite.* (2019) 140:248–54. 10.1016/j.appet.2019.05.031 31141706 PMC11490937

[3] Roy SK Jahan K Alam N Rois R Ferdaus A Israt S Perceived stress, eating behavior, and overweight and obesity among urban adolescents. *J Health Popul Nutr.* (2021) 40:54. 10.1186/s41043-021-00279-2 34920764 PMC8679564

[4] MacLean PS Wing RR Davidson T Epstein L Goodpaster B Hall KD Nih working group report: innovative research to improve maintenance of weight loss. *Obesity (Silver Spring).* (2015) 23:7–15. 10.1002/oby.20967 25469998 PMC5841916

[5] Sadowska J Dziaduch I Bruszkowska M Ziółkowska K. Bmi, body perception, and approach to eating and diet in adolescent girls. *SAGE Open.* (2020) 10:2158244020962816. 10.1177/2158244020962816

[6] Barrera M Jr. Toobert DJ Angell KL Glasgow RE Mackinnon DP. Social support and social-ecological resources as mediators of lifestyle intervention effects for type 2 diabetes. *J Health Psychol.* (2006) 11:483–95. 10.1177/1359105306063321 16774900

[7] AbuSabha R Achterberg C. Review of self-efficacy and locus of control for nutrition- and health-related behavior. *J Am Diet Assoc.* (1997) 97:1122–32. 10.1016/s0002-8223(97)00273-3 9336559

[8] van den Enden G Geyskens K Goukens C. Feeling well surrounded: perceived societal support fosters healthy eating. *J Health Psychol.* (2024) 29:113–22. 10.1177/13591053231178093 37338136 PMC10799535

[9] HaidarARanjitNSaxtonDHoelscherDM. Perceived parental and peer social support is associated with healthier diets in adolescents. *J Nutr Educ Behav.* (2019) 51:23–31. 10.1016/j.jneb.2018.10.003 30635106

[10] Tomás CC Oliveira E Sousa D Uba-Chupel M Furtado G Rocha C Proceedings of the 3rd Ipleiria’s International Health Congress : Leiria, Portugal. 6-7 May 2016. *BMC Health Serv Res.* (2016) 16(Suppl. 3):200. 10.1186/s12913-016-1423-5 27409075 PMC4943498

[11] Smith ML Lee S Towne SD Han G Quinn C Peña-Purcell NC Impact of a behavioral intervention on diet, eating patterns, self-efficacy, and social support. *J Nutr Educ Behav.* (2020) 52:180–6. 10.1016/j.jneb.2019.06.008 31540863

[12] Su D Wu XN Zhang YX Li HP Wang WL Zhang JP Depression and social support between China’ rural and urban empty-nest elderly. *Arch Gerontol Geriatr.* (2012) 55:564–9. 10.1016/j.archger.2012.06.006 22776885

[13] Yang L Li K Liang Y Zhao Q Cui D Zhu X. Mediating role diet self-efficacy plays in the relationship between social support and diet self-management for patients with type 2 diabetes. *Arch Public Health.* (2021) 79:14. 10.1186/s13690-021-00533-3 33517902 PMC7849071

[14] Park Y Kim J. Development and effect of child obesity management program by applied nudge. *Int J Environ Res Public Health.* (2022) 19:12692. 10.3390/ijerph191912692 36231990 PMC9566519

[15] Groshon LC Pearl RL. Longitudinal associations of binge eating with internalized weight stigma and eating self-efficacy. *Eat Behav.* (2023) 50:101785. 10.1016/j.eatbeh.2023.101785 37515998 PMC10839945

[16] Salles A. Self-efficacy as a measure of confidence. *JAMA Surg.* (2017) 152:506–7. 10.1001/jamasurg.2017.0035 28273296

[17] Romano E Haynes A Robinson E. Erratum: weight perception, weight stigma concerns, and overeating. *Obesity (Silver Spring).* (2019) 27:1207–9. 10.1002/oby.22515 31231961 PMC6885919

[18] Zullig KJ Matthews-Ewald MR Valois RF. Weight perceptions, disordered eating behaviors, and emotional self-efficacy among high school adolescents. *Eat Behav.* (2016) 21:1–6. 10.1016/j.eatbeh.2015.11.007 26697720

[19] Butcher NJ Monsour A Mew EJ Chan AW Moher D Mayo-Wilson E Guidelines for reporting outcomes in trial reports: the consort-outcomes 2022 extension. *JAMA.* (2022) 328:2252–64. 10.1001/jama.2022.21022 36511921

[20] Vaux A. Appraisals of social support: love, respect, and involvement. *J Commun Psychol.* (1987) 15:493–502. 10.1002/1520-6629(198710)15:43.0.CO;2-4

[21] Lu Y Yang D Niu Y Zhang H Du B Jiang X. Factors associated with the resilience of Tibetan adolescent survivors five years after the 2010 Yushu earthquake. *PLoS One.* (2020) 15:e0231736. 10.1371/journal.pone.0231736 32324755 PMC7179896

[22] Clark MM Abrams DB Niaura RS Eaton CA Rossi JS. Self-efficacy in weight management. *J Consult Clin Psychol.* (1991) 59:739–44. 10.1037//0022-006x.59.5.739 1955608

[23] Ames GE Heckman MG Grothe KB Clark MM. Eating self-efficacy: development of a short-form wel. *Eat Behav.* (2012) 13:375–8. 10.1016/j.eatbeh.2012.03.013 23121791

[24] Flølo TN Tell GS Kolotkin RL Aasprang A Norekvål TM Våge V Eating self-efficacy as predictor of long-term weight loss and obesity-specific quality of life after sleeve gastrectomy: a prospective cohort study. *Surg Obes Relat Dis.* (2019) 15:161–7. 10.1016/j.soard.2018.12.011 30709748

[25] Killen JD Taylor CB Hayward C Wilson DM Haydel KF Hammer LD Pursuit of thinness and onset of eating disorder symptoms in a community sample of adolescent girls: a three-year prospective analysis. *Int J Eat Disord.* (1994) 16:227–38. 10.1002/1098-108x(199411)16:33.0.co;2-l 7833956

[26] Brasil KM Mims CE McDermott RC Pritchard ME. Checking the scales: a psychometric evaluation of the weight concerns scale in a sample of college-aged cisgender men from the United States. *Psychol Assess.* (2023) 35:218–28. 10.1037/pas0001198 36455029

[27] China C. *Comprehensive Report of the Chinese Adolescent Health-Related and Risk Behaviour Survey.* Beijing: Peking University Medical Press (2007).

[28] Hayes AF. An index and test of linear moderated mediation. *Multiv Behav Res.* (2015) 50:1–22. 10.1080/00273171.2014.962683 26609740

[29] Spiller SA Fitzsimons GJ Lynch JG Mcclelland GH. Spotlights, floodlights, and the magic number zero: simple effects tests in moderated regression. *J Market Res.* (2013) 50:277–88. 10.1509/jmr.12.0420 11670861

[30] Ovaskainen ML Tapanainen H Laatikainen T Mannisto S Heinonen H Vartiainen E. Perceived health-related self-efficacy associated with Bmi in adults in a population-based survey. *Scand J Public Health.* (2015) 43:197–203. 10.1177/1403494814566263 25586112

[31] Silva WRD Santana MS Maroco J Maloa BFS Campos J. Body weight concerns: cross-national study and identification of factors related to eating disorders. *PLoS One.* (2017) 12:e0180125. 10.1371/journal.pone.0180125 28686602 PMC5501473

[32] Ng JY Ntoumanis N Thogersen-Ntoumani C Deci EL Ryan RM Duda JL Self-determination theory applied to health contexts: a meta-analysis. *Perspect Psychol Sci.* (2012) 7:325–40. 10.1177/1745691612447309 26168470

[33] Annesi JJ Gorjala S. Relations of self-regulation and self-efficacy for exercise and eating and Bmi change: a field investigation. *Biopsychosoc Med.* (2010) 4:10. 10.1186/1751-0759-4-10 20815891 PMC2941739

[34] Smith AD Sanchez N Harrison K Bourne C Clark ELM Miller RL Observations of parent-adolescent interactions relate to food parenting practices and adolescent disordered eating in adolescents at risk for adult obesity. *Fam Process.* (2023) 62:1687–708. 10.1111/famp.12829 36347267 PMC11045300

[35] Oudekerk BA Allen JP Hessel ET Molloy LE. The cascading development of autonomy and relatedness from adolescence to adulthood. *Child Dev.* (2015) 86:472–85. 10.1111/cdev.12313 25345623 PMC4376599

